# Feasibility, Acceptability, and Effectiveness of a Smartphone App to Increase Pretransplant Vaccine Rates: Usability Study

**DOI:** 10.2196/68855

**Published:** 2025-04-15

**Authors:** Amy G Feldman, Brenda L Beaty, Susan L Moore, Sheana Bull, Kumanan Wilson, Katherine M Atkinson, Cameron Bell, Kathryn M Denize, Allison Kempe

**Affiliations:** 1 Section of Pediatric Gastroenterology, Hepatology, and Nutrition, University of Colorado, 13123 E 16th Ave, Aurora, CO, 80045, United States, 1 720-777-5354; 2 Digestive Health Institute, Children's Hospital Colorado, Aurora, CO, United States; 3 Adult and Child Center for Outcomes Research and Delivery Science, Aurora, CO, United States; 4 Colorado School of Public Health, Aurora, CO, United States; 5 Department of Medicine, University of Ottawa, Ottawa, ON, Canada; 6 Bruyère Health Research Institute, Ottawa, ON, Canada; 7 CANImmunize Inc, Ottawa, ON, Canada; 8 Department of Global Public Health, Karolinska Institutet, Stockholm, Sweden; 9 Children's Hospital Colorado, Aurora, CO, United States

**Keywords:** mobile app, vaccines, immunizations, transplantation, children, beta test, mobile health, mHealth

## Abstract

**Background:**

Vaccine-preventable infections result in significant morbidity, mortality, and costs in pediatric transplant recipients. Despite intensive medical care in the pretransplant period, less than 20% of children are up to date for age-appropriate vaccines at the time of transplant. Mobile health apps have the potential to improve pretransplant vaccine rates.

**Objective:**

This paper aimed to perform phase 2 beta testing of the smartphone app, Immunize PediatricTransplant*,* to determine (1) if it was effective in achieving up-to-date vaccine status by the time of transplant in a cohort of children awaiting transplants and (2) if the app was feasible and acceptable to parent and transplant provider users.

**Methods:**

We recruited 25 dyads of parents and providers of a child awaiting a liver, kidney, or heart transplant at Children’s Hospital Colorado, Ann and Robert H. Lurie Children’s Hospital, and the Children’s Hospital of Philadelphia. Parents and providers filled out an entry questionnaire before app use to gather baseline information. A research team member entered the child’s vaccine records into the app. The parent and provider downloaded and used the app until the transplant to view vaccine records, read vaccine education, communicate with team members, and receive overdue vaccine reminders. After the transplant (or on April 1, 2024, the conclusion of the study), the parent and provider filled out an exit questionnaire to explore feasibility and acceptability of the app. The child’s vaccine records were reviewed to determine if the child was up to date on vaccines at the time of transplant.

**Results:**

Twenty-five parent and provider dyads were enrolled; 56% (14/25) had a child awaiting a liver transplant, 28% (7/25) had a child awaiting a kidney transplant, and 16% (4/25) had a child awaiting a heart transplant. At the conclusion of the study, 96% (24/25) of the children were up to date on vaccines. Of the 36 parents and providers who filled out an exit questionnaire, 97% (n=35) agreed or strongly agreed that they felt knowledgeable about pretransplant vaccine use and 86% (n=31) agreed or strongly agreed that communication around vaccines was good after using the app. Further, 91% (20/22) of parents and 79% (11/14) of providers recommended the app to future parents and providers of transplant candidates. Parents and providers suggested that in the future the app should connect directly to the electronic medical record or state vaccine registries to obtain vaccine data.

**Conclusions:**

The overwhelming majority of children whose parents and providers used the Immunize PediatricTransplant app were up to date on vaccines at the time of transplant. The majority of app users felt the app was feasible and acceptable. In future iterations of the app and subsequent clinical trials, we will explore whether application programming interfaces might be used to extract vaccine data from the electronic medical record. If implemented broadly, this app has the potential to improve pretransplant vaccine rates, resulting in fewer posttransplant infections and improved posttransplant outcomes.

## Introduction

Transplant recipients are on life-long immunosuppressive medications to prevent graft rejection. As a result of these medications, they are at increased risk for life-threatening infections including vaccine-preventable infections (VPIs) [1-3]. In the first 5 years posttransplant, VPIs occur in pediatric solid organ transplant recipients at rates up to 87 times higher than in the general pediatric population [4,5]. VPIs result in significant morbidity, mortality, and increased hospitalization costs [4,5]. Pretransplant vaccines are an important strategy to decrease the risk of VPIs posttransplant. Unfortunately, despite intensive medical management in the pretransplant period, less than 20% of pediatric liver transplant recipients have received all age-appropriate vaccines by the time of transplant [6]. Barriers to pretransplant vaccination include gaps in knowledge about pretransplant vaccines, lack of communication between team members regarding vaccines, difficulty remembering when vaccines are due amidst other acute medical problems, and lack of an easily accessible centralized vaccine record [7].

Mobile health (mHealth) apps can assist with medical education, team communication, and patient adherence [8-12]. Specifically, mHealth apps have demonstrated utility in facilitating vaccine delivery in the general population [13-20]. In a previous study, our team developed a novel mHealth app, Immunize PediatricTransplant, to help improve vaccine rates in the pretransplant period [21]. Specifically, the content of the app (1) provides information about vaccines in the pretransplant period; (2) houses a cloud-based, easily accessible vaccine record; (3) includes a chat and communication feature to facilitate communication between parents and transplant providers about vaccines; and (4) provides text or email reminders for parents and providers when vaccines are overdue [21].

**Figure 1.**
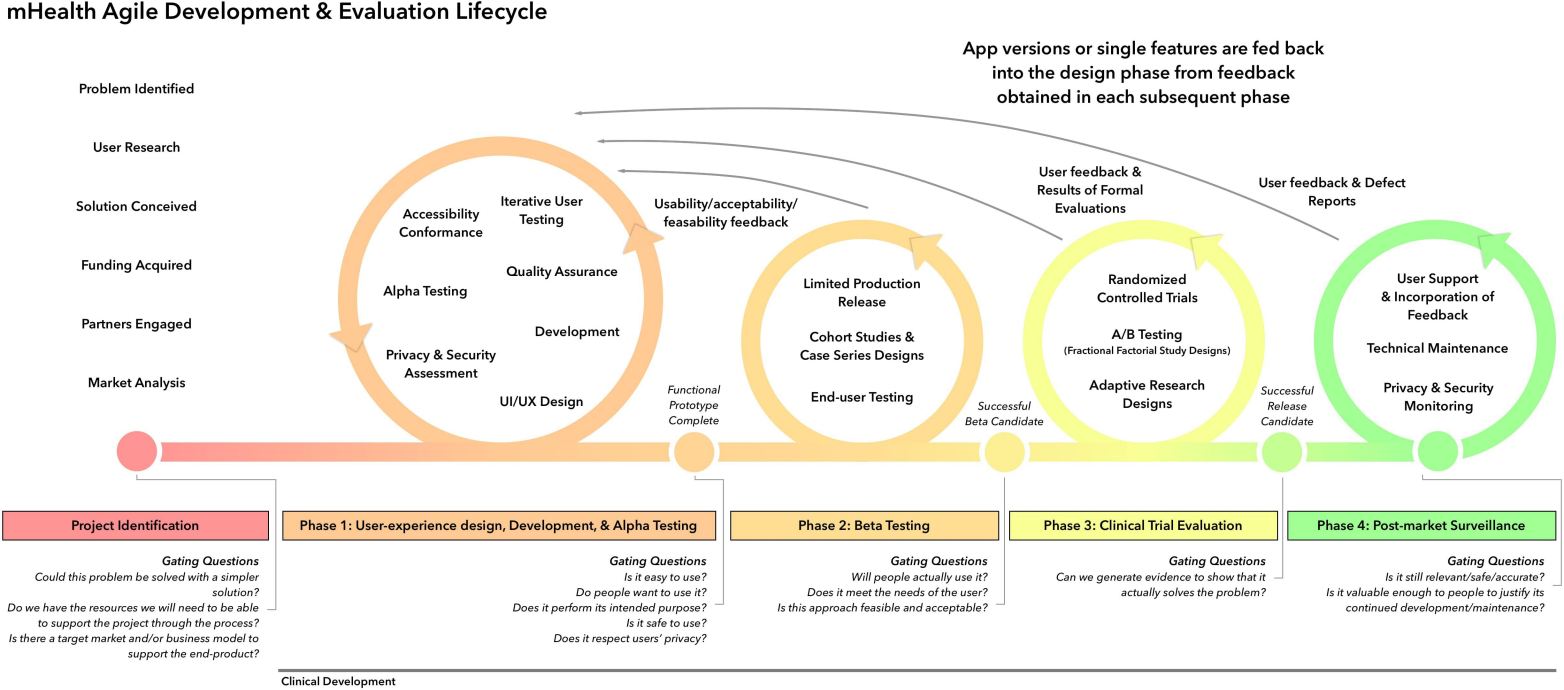
mHealth Agile Development and Evaluation Lifecycle (adapted from Wilson et al [22]). mHealth: mobile health.

According to the mHealth Agile Development and Evaluation Lifecycle (Figure 1) [22], after a functional mobile tool is developed, phase 2 beta testing is needed to test the mHealth product amongst external users to understand if the prototype meets the needs of users in a way that will lead to sustained adoption. Therefore, we designed this study to (1) evaluate the effectiveness of the Immunize PediatricTransplant app in achieving up-to-date vaccine status by the day of transplant (or conclusion of the study on April 1, 2024) and (2) explore app feasibility and acceptability amongst parent and provider users.

## Methods

### Study Design, Participants, and Recruitment

This prospective cohort study was conducted with enrollment between November 1, 2021, and July 31, 2023. Dyads of parents and transplant providers caring for a child awaiting a heart, liver, or kidney transplant were recruited from the Children’s Hospital Colorado, Ann and Robert H. Lurie Children’s Hospital, and the Children’s Hospital of Philadelphia via an in-clinic, email, or electronic medical record (EMR) invitation. Parents of a child being transplanted for acute organ failure or retransplantation were excluded. Parents who refused vaccines for religious or ethical reasons, who were not fluent in English, or who did not have access to a mobile phone were excluded. Certain transplant providers had more than 1 patient enrolled in the study and used the app for all enrolled patients. All participants received a US $100 gift card for participation.

The primary outcome of this study was up-to-date vaccine status of the child on the day of transplant (or end of the study) according to standard Centers for Disease Control (CDC) vaccine recommendations [23]. Secondary outcomes included feasibility and acceptability of the app amongst parent and provider app users.

### Study Procedures and Data Collection

#### Getting to Know You Entry Questionnaire

Each parent and transplant provider were given a baseline survey to gather information about them.

#### Immunize PediatricTransplant App

After the Getting to Know You questionnaire was completed, the parent and provider both downloaded the app. A research team member entered the child’s vaccine records (using state registries, the EMR, and physician and parental records) to initiate the app to send text and email reminders to the parents and providers when a vaccine was due. Parents and providers were instructed to log into the app as often as they wished to view their child’s vaccine records, read information about vaccines, or communicate with team members about vaccines. Vaccine reminders were sent to parents and transplant providers (via text, email, and app notification) when vaccines were due starting at enrollment and going until the time of transplant or end of the study.

#### Exit Questionnaire

After the transplant, the parent and provider filled out a survey with questions assessing app feasibility (How many months did you use the app for? Did you experience technical difficulties with the app?) and acceptability (Would you recommend the app to others? Did you feel communication between families and providers around vaccines was good after using the app? Did you feel knowledgeable about pretransplant vaccines after app use?). The exit questionnaire included an open-ended question asking for suggestions for future app improvement. The exit questionnaire was sent via email after the transplant was completed. Weekly reminders were sent until the questionnaire was complete.

#### Vaccine Rates at the Time of Transplant

A research team member collected vaccine records at the time of transplant to evaluate whether the child was up to date on age-appropriate vaccines at the time of transplant.

### Statistical Analysis

#### Effectiveness

We calculated the percentage of transplant recipients who were completely up to date on age-appropriate vaccines at the time of transplant according to standard CDC vaccine recommendations [23].

#### Feasibility

Data from feasibility questions in the exit questionnaire were summarized through the proportion of the feasibility outcome.

#### Acceptability

Data from acceptability questions in the exit questionnaire were summarized through the proportion of the acceptability outcome.

### Ethical Considerations

The study was approved by the Colorado Multiple Institutional Review Board (CO-18‐0045). All participants provided consent for study participation. Participation was voluntary. An email and password were required to log in to the study app. Participants could access all study content through a mobile app available on both the iOS and Android platforms. All study data were encrypted at rest and in transit. Only authorized researchers were provided access to the participant data. The application’s data was stored exclusively on Amazon Web Services in the United States. Industry standard information security practices were leveraged in the development of the app. All data were deidentified to ensure participant privacy and confidentiality. Participants who completed the full study received US $100 compensation. No artificial intelligence was used in any portion of the manuscript writing.

## Results

### Participants

We enrolled 25 dyads of parents and guardians of heart, liver, and kidney transplant recipients and their transplant provider. The majority (15/25, 60%) of children were <5 years old (Tables1  2).

**Table 1.** Parent and guardian demographic characteristics (n=25).

Characteristic	Value, n (%)
Gender identity	
Female	20 (80)
Male	5 (20)
Ethnicity	
Caucasian	18 (72)
African American	2 (8)
Asian	2 (8)
Hispanic-Latino	1 (4)
Other or prefer not to answer	2 (8)
Center of care	
Children’s Hospital Colorado	19 (76)
Children’s Hospital of Philadelphia	3 (12)
Ann and Robert H. Lurie Children’s Hospital	3 (12)
How far do you live from your transplant center?	
<1 hour drive	8 (32)
1‐ to 3-hour drive	10 (40)
>3-hour drive	3 (12)
We are in a different state than our center and need to fly to our center	4 (16)
How would you describe the town you live in?	
Rural	7 (28)
Suburban	13 (52)
Urban	5 (20)
How old is your child?	
0‐11 months	4 (16)
12 months to 5 years	11 (44)
6‐11 years	4 (16)
12‐18 years	6 (24)
What organ is your child listed for?	
Heart	4 (16)
Liver	14 (56)
Kidney	7 (28)

**Table 2.** Provider demographic characteristics (n=15; there were providers who had multiple patients in the study).

Characteristic	Value, n (%)
Role on transplant team	
Cardiologist, hepatologist, or nephrologist	4 (27)
Transplant fellow	1 (7)
Transplant nurse coordinator	10 (67)
What transplant center do you work with?	
Children’s Hospital Colorado	10 (67)
Children’s Hospital of Philadelphia	2 (13)
Ann and Robert H. Lurie, Children’s Hospital	3 (20)
How long have you worked as an attending transplant physician, transplant fellow, or transplant nurse coordinator?	
<1 year	3 (20)
1‐5 years	5 (36)
6‐10 years	3 (20)
>10 years	3 (20)
Gender identity	
Female	14 (93)
Male	1 (7)
Ethnicity	
Caucasian	11 (73)
African American	0 (0)
Asian	1 (7)
Hispanic-Latino	1 (7)
Other or prefer not to answer	2 (13)

### Effectiveness of the App

Vaccine records were obtained on the day of transplant; 96% (24/25) of children were up to date on all age-appropriate vaccines. The 1 patient who was not up to date was a 15-year-old who was missing a second human papillomavirus (HPV) shot. The patient’s team had specifically chosen to delay the second HPV vaccine until after transplant because the patient was on high-dose steroids for autoimmune hepatitis and was unlikely to mount a strong immune response to a pretransplant vaccine.

### Feasibility of the App

#### App Usage

Parents and providers were instructed to use the Immunize PediatricTransplant app until the day of their child’s transplant (or until the end of the study on April 1, 2024, whichever came first). No children were transplanted in less than a month, 27% (6/22) were transplanted in 1‐3 months, 23% (5/22) in 4‐6 months, and 50% (11/22) in 7 or more months.

#### Technical Difficulties While Using the App

Only 23% (5/22) of parents and 21% (3/14) of providers reported technical difficulties with the app. Difficulties described included initial log on (n=5), entry of historical vaccines or vaccines given while using the app (n=2), and communication with team members through the portal (n=1).

### Acceptability of the App

Of the 25 parents and guardians and 15 providers, 88% (22/25) and 93% (14/15), respectively, completed an exit questionnaire. Of these, 97% (35/36) agreed or strongly agreed that they felt knowledgeable about vaccine use in the pretransplant period after using the app and 86% (31/36) agreed or strongly agreed that communication around vaccines between families and providers was good after using the app. Users rated the following features as extremely or moderately useful: the centralized vaccine record (n=30, 83%), the automated vaccine reminders (n=24, 67%), and the written educational information (n=21, 58%; Table 3). Further, 91% (20/22) of parents and 79% (11/14) of providers recommended the app to future families and transplant teams with children awaiting a transplant.

**Table 3.** Acceptability of the various features of the app (n=36; parents, guardians, and providers combined).

	Extremely or moderately useful, n (%)	Somewhat useful, n (%)	Not useful, n (%)
Written educational information in the app	21 (58)	12 (33)	3 (8)
Communication tool (chat feature)	18 (50)	9 (25)	9 (25)
Automated vaccine reminders	24 (67)	8 (22)	4 (11)
Centralized vaccine record	30 (83)	2 (6)	4 (11)

### Suggestions for App Improvement

The open-ended responses to the question of “Do you have any suggestions for app improvement?” are listed in Textbox 1. The most common suggestion (n=6) was to populate the vaccine history from the EMR or other vaccine databases such as immunization information systems (IISs).

Textbox 1. **Open-ended responses in the exit questionnaire to “Do you have any suggestions for app improvement?”**

**Responses**

- Pull vaccine records from electronic medical record
- Automatically populate the vaccination history
- When entering vaccines, it would be nice to enter more than one at a time
- Scan vaccine documents or have a professional health care provider enter in the vaccine data, not the family, there’s too much room for error
- Allow downloads of vaccine records from state immunization information systems (IIS)
- Connect the app to a vaccine data base
- Space for additional vaccines (extra COVID doses or repeat series)
- Make the live chat a more noticeable feature
- Improve ease of use for vaccine entry
- Connect primary care provider with the app
- Have a way to submit option of family refusal to avoid getting reminders about a vaccine your family is refusing
- Tech support for users
- Create a way for medical providers to silence alerts for vaccines that family is refusing
- App appearance
- If we could have the primary care pediatrician participate, that would be great
- Encourage primary care pediatricians to use it for communication
- Integration with vaccine databases such as the Colorado Department of Public Health and Environment

## Discussion

### Principal Findings

We sought to beta test the Immunize PediatricTransplant mHealth app to understand whether (1) it could help address the problem of underimmunization of pediatric solid organ transplant candidates and (2) it would be feasible and acceptable to parent and transplant provider users. The most significant finding of the study is the success of the app in getting nearly all children up to date on age-appropriate vaccines by the time of transplant. Historically, the majority of pediatric transplant candidates have not been up to date on age-appropriate vaccines at the time of transplant. In a study of 281 children who underwent a pediatric liver transplant across the United States between August 2017 and August 2018, only 19% were up to date on age-appropriate vaccines on the day of transplant and amongst those who were not up to date, 51% were missing 4 or more immunizations [6]. Increasing immunization rates pretransplant could significantly improve posttransplant outcomes by decreasing posttransplant VPIs and the associated morbidity, mortality, and increased medical costs [4,5].

While the app was overall feasible and acceptable to users (31/36, 86% of users recommended the app to future parents and providers), in the open-ended question in the exit questionnaire asking for suggestions for app improvement we learned that users did not want to have to enter vaccine records themselves into the app (in real-world use, there would be no research coordinator to enter vaccines). Multiple users recommended that the app connect directly with the EMR or IISs to retrieve accurate vaccine data.

There are several apps currently available in the App Store to promote childhood vaccination [24]. To our knowledge, Immunize PediatricTransplant is the first app designed specifically for a group of children with complex medical needs at a higher risk for acquiring VPIs. In a 2020 systemic review of 28 studies evaluating 25 immunization apps, 4 studies described significant benefits of implementation of the app and 4 studies reported a significant impact on knowledge and vaccine decision-making [25]. Our study adds additional evidence that an mHealth app can improve vaccine rates in a high-risk population of children. In addition to providing vaccine education, record keeping, and reminder systems, Immunize PediatricTransplant also includes a communication portal for families and providers to directly communicate in real time about vaccine questions.

### Future Directions

Based on user feedback, in the future we will explore utilizing application programming interfaces (APIs) like Health Level 7 FHIR (Fast Healthcare Interoperability Resources) [26] and SMART on FHIR [27] to allow the app to directly connect and communicate with the EMR and IISs [28]. While APIs are legal according to the 21st Century Cures Act [29], it would be critical to ensure compliance with Health Insurance Portability and Accountability Act (HIPAA) regulations protecting patient privacy. We will then need to evaluate the tool in a clinical trial (phase 3 of the mHealth Agile Development and Evaluation Lifecycle) [22] to see if it results in up-to-date immunization status at the time of transplant for a diverse population of pediatric transplant candidates across the country. As this is a “low-risk app” that does not pose any risk to user safety if it does not function (providers should still be reviewing vaccines as part of standard medical care), an alternative to a formal clinical trial would be ongoing A/B testing where a group of users trial a new version of the app that contains minor variations to a specific component of the app [22]. Based on user feedback, ongoing iterations of the app could be made. In future studies, we will modify the primary end point to not classify patients with deliberately missed vaccines (as instructed by the medical team) as not up to date.

After clinical trial or A/B testing, postmarket surveillance will be important to gather ongoing feedback about if the app remains useful and effective and whether further improvements are needed to keep it relevant as new vaccines come into the market [22]. In future research, it will be important to expand the qualitative sections of the exit questionnaire or perform postintervention interviews or focus groups to gain a deeper understanding of the users’ experiences, likes, and dislikes about the app and recommendations for future app users including primary care physicians.

### Limitations

Multiple limitations exist in this study beta testing the Immunize PediatricTransplant app. Our sample size was small (only 25 dyads of parents and transplant providers, which equated to 36 app users) and the majority of participants came from 1 large academic pediatric transplant center. Additionally, the app was only trialed by English-speaking parents and providers, limiting generalizability to all transplant parents and providers. Participation in the study was voluntary, which could introduce selection bias where people who were more interested in or comfortable with mHealth technology were more likely to participate. Similarly, there could be social desirability bias, where people were more likely to report finding the app to be beneficial in an attempt to please researchers. Finally, it is possible that simply by having parents and providers participate in an intervention-based research study, it increased attention and awareness to vaccines, in turn increasing the amount of transplant recipients with up-to-date vaccine statuses. As this was not a randomized trial, there was no control group to compare with. These limitations are all inherent to beta testing, and the app will need to go through future phase 3 clinical trial evaluation.

### Conclusions

Each year, the United States invests $1.2 billion dollars in pediatric solid organ transplants [30]; however, we fail to protect our investment by not ensuring that these children are fully immunized. A novel mobile app, Immunize PediatricTransplant, has now been developed to help overcome barriers to pretransplant vaccines. The app was feasible and acceptable to parent and provider users and successfully resulted in nearly all children being up to date on age-appropriate vaccines by the time of transplant. However, the app was reliant on a research team member entering historical vaccine information, which is not practical for real-world use. In future iterations, we will need to explore whether APIs could be leveraged to extract vaccine information from the EMR or IISs and directly input these data into the app.

## Abbreviations:

API: application programing interface
CDC: Centers for Disease Control
EMR: electronic medical record
FHIR: Fast Healthcare Interoperability Resources
HIPAA: Health Insurance Portability and Accountability Act
HPV: human papillomavirus
IIS: immunization information system
mHealth: mobile health
VPI: vaccine-preventable infection
